# Biparametric Prostate MRI: Diagnostic Performance, Estimated Additional Cost of DCE, and Gadolinium Avoidance

**DOI:** 10.3390/jimaging12100470

**Published:** 2026-09-27

**Authors:** Gian Carlo Parenti, Nicola Pegoraro, Aldo Carnevale, Emidio Colleluori, Marcello Pasqualini, Lorenzo Mellini, Giuseppe Falcone, Francesco Feletti

**Affiliations:** 1 Unità Operativa di Radiologia, Ospedale S. Maria delle Croci, Ravenna, Azienda USL della Romagna, 48121 Ravenna, Italy; 2 Dipartimento di Medicina Traslazionale, Università di Ferrara, 44121 Ferrara, Italy; 3 Unità Operativa Radiologia Universitaria, Azienda Ospedaliero-Universitaria “S. Anna”, 44124 Ferrara, Italy

**Keywords:** healthcare management, one health, cancer, biparametric MRI, aquatic pollution, gadolinium-based contrast agents

## Abstract

Biparametric MRI, which excludes dynamic contrast-enhanced imaging, may reduce the resources required for prostate MRI. This exploratory study assessed biparametric MRI findings against biopsy histopathology in a selected cohort and described differences from the original clinical multiparametric MRI readings. A retrospective convenience sample of 89 prostate MRI examinations with an available biparametric reassessment after removal of dynamic contrast-enhanced sequences was identified. Four patients without a classifiable histopathological diagnosis were excluded, leaving 85 patients for analysis. All had undergone biopsy following a suspicious clinical multiparametric MRI report. Two experienced radiologists each reviewed a portion of the biparametric MRI examinations while blinded to histopathology and the original multiparametric MRI report. Clinically significant prostate cancer, defined as Gleason score ≥3 + 4 (Grade Group ≥ 2), was the primary outcome. PI-RADS > 3 was considered positive. Prostate cancer was detected in 47 of 85 patients (55.3%), including 34 (40.0%) with clinically significant disease. Biparametric MRI classified 33 of 34 clinically significant cancers as PI-RADS > 3, corresponding to a sensitivity of 97.1% (95% CI, 85.1–99.5%). The corresponding sensitivity of the original clinical multiparametric MRI readings was 91.2% (95% CI, 77.0–97.0%; exploratory paired comparison, *p* = 0.50). Omitting dynamic contrast-enhanced imaging was estimated to reduce examination time, administered gadolinium, and the use of contrast-related materials and staff capacity under local workflow assumptions. In this selected biopsy cohort, biparametric MRI classified 33 of 34 clinically significant cancers as PI-RADS > 3. The comparison with the original multiparametric MRI readings was exploratory and cannot establish equivalence or non-inferiority. Avoiding dynamic contrast-enhanced imaging may reduce contrast administration and resource use, although the economic estimates are specific to the local workflow and environmental release was not measured.

## 1. Introduction

Biparametric magnetic resonance imaging (bpMRI) of the prostate, performed without the dynamic contrast-enhanced (DCE) sequence, is being investigated as an alternative to multiparametric MRI (mpMRI) for detecting clinically significant prostate cancer (csPCa). Indeed, omitting gadolinium-based contrast agents (GBCAs) reduces examination time and patient discomfort, both of which are often cited as major limitations of mpMRI [1,2].

Since PI-RADS version 2 (2015), DCE has had a secondary role [3]. Studies have subsequently reported comparable diagnostic performance for bpMRI based on T2-weighted and diffusion-weighted imaging across different clinical settings [4,5]. This approach may preserve diagnostic performance while reducing resource use and environmental impact.

However, avoiding the DCE sequence may have further implications that should be investigated.

Gadolinium administered during contrast-enhanced MRI is excreted and may enter aquatic environments, where increasing anthropogenic concentrations have been reported [6]. Separate concerns relate to gadolinium retention and possible adverse effects after GBCA exposure, although their clinical significance remains uncertain [7,8,9].

The reduction in examination duration may be beneficial in an era when the growing demand for prostate MRI examinations raises concerns about the limited scanner time available on suitably equipped MRI systems to meet this demand and the shortage of qualified radiologists, technicians, and nursing staff [10].

This exploratory study assessed bpMRI findings against biopsy histopathology in a selected biopsy cohort and described differences from the original clinical mpMRI readings. It also estimated the additional local resource use attributable to the DCE workflow and the amount of gadolinium administration that would be avoided by omitting DCE.

## 2. Materials and Methods

During the study period (2020–2023), 250 prostate MRI examinations were recorded in the institutional source database. Retrospective bpMRI readings were available for 89 examinations at database closure; these examinations therefore formed a convenience sample rather than a formally randomized sample. Selection was not based on clinical characteristics, imaging findings, or histopathological outcomes. Three patients were excluded because histopathological data were unavailable and one because the histopathological outcome could not be classified, leaving 85 patients for the final analysis. All included patients had undergone biopsy following a clinical mpMRI report considered suspicious for malignancy. The study was approved by the Comitato Etico della Romagna (CET-CEROM; study registration no. 4085; protocol no. 1308/2026, I.5/27). All MRI examinations were performed on a 1.5-T scanner (Achieva, Philips Healthcare, Best, Netherlands) using a surface coil (Philips Sense Flex Medium coil) and a standardised protocol. Patients were positioned supine, feet first, in the scanner. For mpMRI examinations, a peripheral intravenous line was placed in the antecubital vein and connected to a power injector for contrast administration.

The mpMRI protocol included:

- High-resolution T2-weighted sequences acquired in axial, sagittal, and coronal planes (slice thickness: 3 mm);
- Diffusion-weighted imaging (DWI) using a single-shot spin-echo echo-planar sequence with multiple b-values (100, 500, 1000 and 1600 s/mm^2^), with apparent diffusion coefficient (ADC) maps generated using a monoexponential model;
- Dynamic contrast-enhanced (DCE) imaging with T1-weighted sequences with a temporal resolution 7.7 s, following intravenous injection of gadolinium-based contrast agent (0.1 mmol/kg), followed by a saline flush.

BpMRI datasets were obtained by removing the DCE series from the original examinations, leaving T2-weighted and diffusion-weighted images. Before starting the retrospective bpMRI assessment, the two radiologists (LM and GF) discussed the application of PI-RADS version 2.1 and jointly reviewed the first examination. The remaining 88 examinations were randomly allocated between the two readers, with 39 examinations assigned to Reader 1 and 49 to Reader 2. Each examination was then interpreted independently by the assigned radiologist. The readers had no access to histopathology or to the original mpMRI images and reports, although they knew that the patients had undergone biopsy. As the cases were not read by both radiologists, inter-reader agreement could not be calculated. BpMRI scores were compared with those in the original clinical mpMRI reports, which were not reassessed under the same blinded conditions; the comparison therefore reflects two different reading settings.

All patients underwent MRI/ultrasound fusion-guided prostate biopsy using an elastic fusion technique (Koelis (TM) system; Figure 1). Biopsy targets were defined from the original clinical mpMRI and sampled through a transrectal approach after real-time fusion with TRUS. Additional non-targeted cores were obtained from other prostate sites during the same biopsy session as part of routine clinical practice but were not considered in the present analysis.

The bpMRI readings were produced retrospectively and did not influence biopsy targeting or mapping. Histopathology was used as the reference standard, but because target selection depended on the original mpMRI, the reference pathway was not independent of that examination. This design may have preferentially verified lesions identified on mpMRI and may have affected estimates of sensitivity and false-negative findings for both protocols.

Staff time evaluation was estimated according to the Emilia-Romagna Region and Italian Society of Medical and Interventional Radiology (SIRM) reference standards [11,12]. For mpMRI, the standard workflow included nurse, radiographer, and radiologist time associated with contrast-enhanced examinations. For bpMRI, only radiographer and radiologist time related to the non-contrast protocol was considered.

The cost analysis took the perspective of the public hospital provider and covered resources attributable to DCE rather than the full cost of mpMRI. Calculations used the mean GBCA volume administered in the cohort (16.34 mL), local purchase prices for contrast medium and consumables, and the additional staff and scanner time associated with the contrast-enhanced workflow. The model assigned 10 min to the radiographer, radiologist, and MRI system and 40 min to nursing activities, including venous access, contrast administration, and readiness to manage adverse reactions. Staff costs were based on the relevant Italian National Collective Labour Agreements (CCNL), while equipment costs comprised depreciation and maintenance per scanner hour. Drug and material prices referred to 2025 and equipment costs to 2024, without adjustment for inflation. Overheads, opportunity costs, treatment of adverse events, and downstream clinical costs were outside the scope of the analysis.

For the environmental assessment, the volume of GBCA administered to each patient was recorded from the MRI reports. The total amount of gadolinium administered was estimated assuming a GBCA concentration of 0.5 mmol/mL and a gadolinium atomic mass of 157.25 g/mol. The cumulative gadolinium load was calculated for the 85 patients included in the final analysis.

### Statistical Analysis

Continuous variables were reported as mean and standard deviation or median and interquartile range, as appropriate; categorical variables were reported as numbers and percentages. The primary outcome was clinically significant prostate cancer, defined as Gleason score ≥3 + 4 (Grade Group ≥ 2), while detection of any cancer was a secondary outcome. Analyses used the highest PI-RADS score recorded for each patient. A score >3 was chosen as the positivity threshold to retain PI-RADS 3 as an equivocal category and to identify examinations that moved into this category after DCE removal. Sensitivity, specificity, positive predictive value, negative predictive value, and accuracy were calculated with 95% Wilson confidence intervals. Paired sensitivities and specificities were compared with exact McNemar tests. AUCs were derived from ordinal PI-RADS scores and compared with DeLong’s test for paired ROC curves; weighted Cohen’s kappa was used to assess agreement between mpMRI and bpMRI scores. All comparisons were exploratory, and a non-significant result was not taken as evidence of equivalence or non-inferiority. Because entry into the cohort followed a suspicious clinical mpMRI report, estimates for mpMRI apply only to this selected biopsy cohort. Tests were two-sided with *p* < 0.05 as the significance threshold. Analyses were performed with Python 3.11 and independently checked by the authors.

## 3. Results

### 3.1. Patient Characteristics

During the study period, 250 prostate MRI examinations were recorded in the institutional source database. Retrospective bpMRI review was available for 89 examinations, which formed a convenience sample. Three patients were excluded because histopathological data were unavailable, and one was excluded because the histopathological outcome could not be classified. The final analysis therefore included 85 biopsied patients selected after a suspicious clinical mpMRI report. Prostate cancer was found in 47 patients (55.3%). Thirty-four patients (40.0%) had clinically significant prostate cancer, and 13 (15.3%) had Gleason 3 + 3 disease (Table 1). These prevalences describe this selected biopsy cohort and should not be extrapolated to the wider population undergoing prostate MRI. The de-identified patient-level data used in the analysis are provided in Table S1.

### 3.2. PI-RADS Scores in mpMRI and bpMRI

On mpMRI, 69/85 examinations (81.2%) had a PI-RADS score > 3; 43 of these patients had cancer. The remaining 16 examinations were PI-RADS 3, and four had a positive biopsy. On bpMRI, 48/85 examinations (56.5%) had a PI-RADS score > 3; 39 of these patients had cancer. Twenty-two examinations were PI-RADS 3, including six positive biopsies. Fifteen examinations were PI-RADS 2, including two Gleason 3 + 3 cancers. Among the 34 patients with clinically significant prostate cancer, 33 had a bpMRI score > 3 (Table 2).

### 3.3. Diagnostic Performance of mpMRI and bpMRI

At a PI-RADS threshold >3, bpMRI identified 33 of the 34 clinically significant cancers and produced 15 false-positive and one false-negative result. The corresponding counts for the original clinical mpMRI reports were 31, 38, and three. Sensitivity was 97.1% for bpMRI (95% CI, 85.1–99.5%) and 91.2% for mpMRI (95% CI, 77.0–97.0%); the exploratory paired comparison gave *p* = 0.50. These figures come from patients selected for biopsy after a suspicious mpMRI report. In particular, no examination scored below PI-RADS 3 on mpMRI entered the cohort: the 16 examinations treated as mpMRI-negative were all PI-RADS 3, whereas 37 examinations were scored PI-RADS ≤ 3 on bpMRI. Table 3 reports the remaining performance measures within this restricted setting. For detection of any prostate cancer, bpMRI sensitivity was 83.0%, specificity 76.3%, and accuracy 80.0%. Agreement between the original mpMRI and retrospective bpMRI scores was moderate (weighted kappa = 0.45).

### 3.4. Staff Time Evaluation

According to the Emilia-Romagna Region and Italian Society of Medical and Interventional Radiology (SIRM) reference standards [11,12], mpMRI requires approximately 40 min, whereas bpMRI requires 30 min (Table 4). Although a radiologist does not need to be present during a non-contrast examination, the estimated radiologist time for bpMRI remains 30 min, including clinical assessment and reporting. Across the 85 patients included in the analysis, omitting DCE corresponded to an estimated reduction of 14.17 h of radiographer time, 14.17 h of radiologist time, and 56.67 h of nursing time. These figures represent potentially reallocatable staff capacity rather than directly observed monetary savings.

### 3.5. Cost Evaluation

The mean volume of contrast agent administered in the 85 patients included in the final analysis was 16.34 mL (range, 12–22 mL), for a total of 1389.1 mL. At a GBCA concentration of 0.5 mmol/mL and a gadolinium atomic mass of 157.25 g/mol, this corresponded to a total of 109.22 g of gadolinium administered across the analyzed cohort.

At our institution, mpMRI examinations are scheduled during dedicated contrast-enhanced imaging sessions, which require the presence of a nurse responsible for venous access placement, contrast injection, and administration of medications in case of adverse reactions.

The additional resource value assigned to DCE was EUR178.86 per examination in the local model. Contrast medium and administration materials accounted for direct expenditure; the remainder represented the value assigned to staff and scanner time. The latter resources might be reassigned if DCE were omitted but would not necessarily produce an equivalent reduction in hospital expenditure. The estimate also depends on local prices, staffing, scanner use, protocol duration, and accounting methods and is not directly transferable to other settings.

This estimate does not take into account the variable amount of saline solution administered for venous access testing and possible pre- or post-examination hydration, nor the cost of medications required in case of adverse events.

The staff, machine, materials and drugs costs included in the estimated additional cost of the contrast-enhanced workflow are summarized in Table 5.

The estimated salary of a mid-level medical consultant was calculated based on the 2019–2021 National Collective Labor Agreement (CCNL) for the Healthcare Area, published on 23 January 2024. The reference profile was that of a full-time medical consultant with twelve years of seniority, positioned in an intermediate pay grade. The estimate included a basic professional assignment and average additional allowances for hospital activities, including night and weekend shifts and on-call duties, as typically required in a 24/7 clinical department.

The estimated salary of a mid-seniority nurse employed in the Italian National Health Service (SSN) was based on the 2019–2021 National Collective Labor Agreement (CCNL) for the Healthcare Sector, published on 2 November 2022. The reference profile was a category D nurse with fifteen years of seniority, positioned in an intermediate pay grade. The estimate included the main additional allowances related to shift and night duties, on-call availability, and overtime on public holidays, consistent with standard hospital practice.

The estimated salary of a mid-seniority radiology nurse was based on the 2019–2021 National Collective Labor Agreement (CCNL) for the Healthcare Sector, published on 2 November 2022. The reference profile was that of a category D nurse with fifteen years of seniority, positioned in an intermediate pay grade. The estimate included the main additional allowances related to shift and night duties, on-call availability, overtime on public holidays, and the radiology allowance, consistent with standard hospital practice.

The depreciation and maintenance costs of the MRI equipment were calculated based on its initial commercial purchase value. Depreciation was analyzed and estimated considering the total number of examinations performed by the Radiology Department of Ravenna in 2024. For maintenance costs, an average rate typically applied in such analyses was adopted, corresponding to 8% of the purchase cost.

## 4. Discussion

Advanced imaging, particularly mpMRI and fusion-guided biopsy, has transformed prostate cancer diagnosis [13,14]. In this biopsy cohort, retrospective bpMRI classified 33 of 34 clinically significant cancers as PI-RADS > 3, compared with 31 of 34 in the original clinical mpMRI reports. The two readings, however, were obtained in different settings: mpMRI was interpreted during clinical care and contributed to the decision to biopsy, whereas bpMRI was reviewed retrospectively without access to the original report or histopathology. The findings are therefore exploratory. Previous studies have examined bpMRI for initial diagnosis [15,16,17], active surveillance [18], and screening [19], mainly as a shorter examination that avoids contrast administration.

The confidence intervals around sensitivity were wide, and the paired comparison was not significant. With only 34 clinically significant cancers and few false-negative examinations, the study cannot determine whether the protocols have equivalent or non-inferior sensitivity. The lower number of false-positive bpMRI classifications is also difficult to interpret because the cohort was selected through mpMRI and contained no mpMRI category below PI-RADS 3.

BpMRI also has limitations. In our series, six more examinations were classified as PI-RADS 3 with bpMRI than with mpMRI (22/85 vs. 16/85), a difference of 7.1 percentage points. Similar findings have been reported [20]. A shift toward PI-RADS 3 increases the number of patients left in an equivocal category and may therefore replace a contrast decision with a management decision rather than simply reducing biopsy use. In practice, the decision to biopsy or monitor such patients cannot be based on the bpMRI score alone. PSA density, clinical risk, previous biopsy results, lesion size and location, patient preference, and local pathways may support either targeted biopsy, interval MRI, or clinical surveillance. The present study was not designed to compare these management strategies or their downstream costs.

Proposed approaches to PI-RADS 3 lesions include additional risk stratification using lesion volume, with a 0.5-mL cutoff [21,22] These approaches require prospective validation and should not be interpreted as universal biopsy thresholds.

DCE may still be useful as a complementary sequence when T2-weighted or DWI image quality is suboptimal and for readers with limited experience [23]. DCE images are not routinely used for MRI/US fusion during targeted biopsy, which relies on T2-weighted and DWI sequences [24].

A small number of PI-RADS 2 lesions on bpMRI were associated with biopsy-proven low-grade cancer. This is consistent with reported PI-RADS 2 outcomes, including a 69.4% probability of negative biopsy, 14.8% of GS 3 + 3, and 15.7% of GS ≥ 7 [25].

### 4.1. Patients’ Safety

BpMRI avoids exposure to gadolinium-based contrast agents. Gadolinium may persist in body tissues and has been associated, although not conclusively, with Gadolinium Deposition Disease (GDD) [9,26,27]. Avoiding repeated contrast administration may be relevant for patients undergoing serial MRI during active surveillance.

### 4.2. Sustainability of the Demand for Diagnostic Services

Demand for prostate MRI is increasing, while scanner time and trained staff remain limited.

In Italy, radiologist and radiologic technologist numbers are close to or above the European average [28], whereas nursing staff remain comparatively scarce.

In 2025, Italy had 5.13 nurses per 1000 inhabitants, compared with an OECD average of 9.8/1000 and a European Union average of 9/1000, with regional values ranging from 3.83 to 7.01 [29]. Under these staffing constraints, bpMRI may reduce staff time as well as the burden and costs associated with contrast administration.

BpMRI can in principle be performed without the resources required for management of severe contrast reactions. However, uncertain examinations may still require DCE [10], reducing this workflow advantage. Any operational benefit therefore needs to be considered together with diagnostic performance [30].

### 4.3. Environmental Aspects

Dekker et al. [6] described dose reduction and non-contrast protocols among the measures available to limit gadolinium release. Applying bpMRI to the present cohort would have avoided 109.22 g of administered gadolinium and shortened acquisition by an estimated 10 min per patient. We did not measure urinary excretion, wastewater concentrations, or ecological effects, so these data quantify avoided administration rather than environmental contamination. The same distinction applies to costs: contrast medium and consumables are direct expenses, whereas released staff and scanner time represent capacity that could be used elsewhere.

### 4.4. Limitations

This retrospective single-center study used a small convenience sample of 89 prostate MRI examinations with available retrospective bpMRI readings, without formal random sampling. The analysis included only patients biopsied after a suspicious clinical mpMRI report. Cancer prevalence is consequently higher than would be expected in the general population referred for prostate MRI. Since the clinical mpMRI findings determined the biopsy targets, lesions visible on mpMRI were more likely to be sampled. Although additional non-targeted cores were obtained during the biopsy session as part of routine clinical practice, these were not considered in the present analysis. The histopathological reference used for the imaging comparison was therefore not independent of the original mpMRI findings, which may have influenced estimates of sensitivity and false-negative findings. The selected nature of the cohort also limits the interpretation of specificity, particularly because no mpMRI examination below PI-RADS 3 was included.

The bpMRI readers reviewed one case together after agreeing on PI-RADS version 2.1, after which the remaining examinations were randomly allocated between them and read by only one radiologist. Consequently, inter-reader reproducibility could not be assessed for bpMRI and was also unavailable for the original clinical mpMRI readings. Recall of individual cases during retrospective review remains possible. Biopsy histology was the reference standard. Sampling may miss a higher-grade component, and some Grade Group 1 tumors could have been classified as clinically significant on prostatectomy. This would change the diagnostic counts, although its extent cannot be estimated because surgical specimens were not available for the entire cohort. Precision was further limited by the 34 clinically significant cancers and the small number of false-negative examinations. The study was exploratory and was not designed as a non-inferiority trial.

The economic analysis reflects local prices and working practices. Staff and scanner time indicate potentially reusable capacity rather than guaranteed cash savings, and downstream costs related to biopsy, surveillance, or repeat MRI in PI-RADS 3 cases were not included. The environmental calculation was similarly confined to gadolinium administration; release into wastewater and ecological effects were not measured.

## 5. Conclusions

BpMRI classified 33 of 34 clinically significant cancers as PI-RADS > 3 in this selected biopsy cohort (sensitivity, 97.1%; 95% CI, 85.1–99.5%). The sample size, cohort selection, and different reading conditions preclude conclusions about equivalence or non-inferiority relative to the original clinical mpMRI reports.

Omitting DCE shortened image acquisition and avoided contrast administration. In the local model, it also removed the cost of GBCA and administration materials and made staff and scanner time available for other activity. These estimates depend on the workflow used at this institution. Likewise, the calculated gadolinium amount concerns administration only and does not quantify release into the environment.

A prospective study should enroll consecutive patients and have both protocols read independently under the same conditions, with a reference standard that is not determined by either imaging arm. Until such data are available, DCE remains useful when image quality is inadequate, lesion assessment is uncertain, or reader experience is limited.

## Figures and Tables

**Figure 1:**
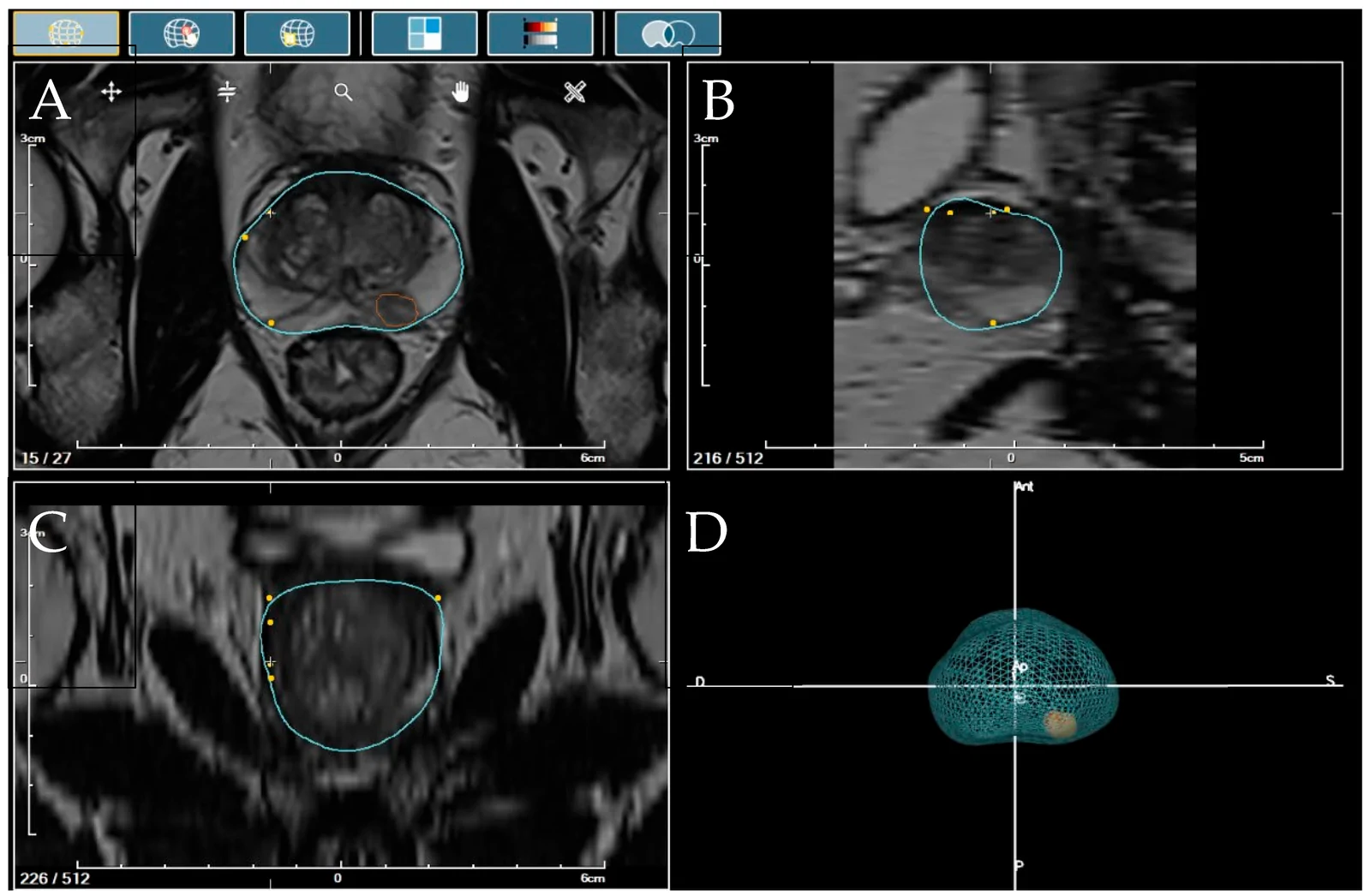
Illustrative example of MRI/ultrasound fusion-guided transrectal prostate biopsy performed using the same fusion platform employed in the study (Koelis™ system), allowing real-time co-registration of transrectal ultrasound (TRUS) and multiparametric MRI (mpMRI) images for targeted sampling. The illustrated case was acquired after the end of the retrospective study period and was not included in the study cohort. The prostate gland is outlined in light blue, while the orange region of interest (ROI) represents the target lesion. (**A**) Axial T2-weighted MRI image showing the target lesion. (**B**) Sagittal T2-weighted MRI reconstruction. (**C**) Coronal T2-weighted MRI reconstruction. (**D**) Three-dimensional reconstruction of the prostate gland and target lesion generated by the fusion platform.

**Table 1:** Patient characteristics and biopsy results.

	All Patients, *n* = 85
Age (years)	Range 49–84, mean 67.2
Positive biopsy (%)	47 (55.3)
GS 6 (%)	13 (15.3)
GS 7a (3 + 4) (%)	14 (16.5)
GS 7b (4 + 3) (%)	7 (8.2)
GS 8 (%)	6 (7.1)
GS 9–10 (%)	7 (8.2)

**Table 2:** PI-RADS classification and biopsy results for mpMRI and bpMRI.

	mpMRI Cases	Positive Biopsy	bpMRI Cases	Positive Biopsy
Total	85	47	85	47
PI-RADS > 3	69	43	48	39
PI-RADS = 3	16	4	22	6
PI-RADS < 3	0	0	15	2

**Table 3:** Diagnostic performance within the selected biopsy cohort, with 95% confidence intervals. Clinically significant prostate cancer was defined as Gleason score ≥3 + 4 (Grade Group ≥ 2), and PI-RADS > 3 was considered positive using the highest patient-level score. Proportions have Wilson confidence intervals. The sensitivity comparison used a two-sided exact McNemar test and is presented as exploratory. Specificity and AUC *p*-values are not reported because cohort selection and unequal reading conditions preclude valid claims of comparative superiority. The mpMRI values derive from the original clinical reports that led to biopsy, and the cohort contained no mpMRI examination below PI-RADS 3. They therefore describe this cohort and are not estimates of performance in an unselected population. Dashes indicate that no inferential comparison was performed.

Parameter	Original Clinical mpMRI (95% CI)	bpMRI (95% CI)
Sensitivity	91.2% (77.0–97.0)	97.1% (85.1–99.5)
Specificity	25.5% (15.5–38.9)	70.6% (57.0–81.3)
PPV	44.9% (33.8–56.6)	68.8% (54.7–80.1)
NPV	81.2% (57.0–93.4)	97.3% (86.2–99.5)
Accuracy	51.8% (41.3–62.1)	81.2% (71.6–88.1)
AUC	0.712 (0.611–0.813)	0.891 (0.828–0.954)

**Table 4:** Standard staff time (expressed in minutes) according to the Italian Society of Medical and Interventional Radiology (SIRM) and the Emilia-Romagna Region guidelines.

	bp-MRI	mp-MRI
Nurse	0	40
Radiographer	30	40
Radiologist	30	40

**Table 5:** Materials, drugs, staff time and machine time required for contrast-enhanced MRI execution. Hydrocortisone and chlorphenamine were available for the management of potential acute hypersensitivity reactions but were not routinely administered; therefore, their cost was not included in the per-examination cost. * Material and drug costs refer to 2025; ** equipment depreciation and maintenance costs refer to 2024. No inflation adjustment was applied to harmonize costs across reference years.

	Acquisition Cost for the Facility (€)	Unit Denominator	Average Unit Cost
Contrast Agent *	87.98	16.34 mL	80.75/15 mL
Sterile Gloves *	0.78	2 units	0.78/2 units
Sterile Gauze 10 × 10 *	0.01	1 unit	0.01/unit
Cannula Needle 20 × 30 *	0.59	1 unit	0.59/unit
Contrast Administration Kit *	26.84	1 unit	26.84/unit
Syringe Kit *	14.64	1 unit	14.64/unit
Saline Solution 500 cc *	0.67	0	0
Hydrocortisone 500 mg *	3.90	0	0
Chlorphenamine Fl *	1.12	0	0
Nurse	25.6 per hour	40 min	17.1 per 40 min
Radiographer	26.2 per hour	10 min	4.4 per 10 min
Radiologist	57.9 per hour	10 min	9.65 per 10 min
Equipment Depreciation and Maintenance Costs **	101.2 per hour	10 min	16.87 per 10 min
Total	-	-	178.86 per exam

## Data Availability

The dataset generated and analyzed in this study are included in the article/Supplementary Material. Further inquiries can be directed to the corresponding author.

## Supplementary Materials

The following supporting information can be downloaded at https://www.mdpi.com/article/10.3390/jimaging12100470/s1. Table S1, de-identified patient-level dataset used for the analyses, including age, mpMRI and bpMRI PI-RADS scores, biopsy outcome, Gleason score, and administered GBCA volume.

## Abbreviations

The following abbreviations are used in this manuscript:

MRI	Magnetic Resonance Imaging
mpMRI	Multiparametric Magnetic Resonance Imaging
bpMRI	Biparametric Magnetic Resonance Imaging
csPCa	Clinically Significant Prostate Cancer
PI-RADS	Prostate Imaging Reporting and Data System
DCE	Dynamic Contrast-Enhanced
GBCA	Gadolinium-Based Contrast Agent
